# *Drosophila* as a model system to unravel the layers of innate immunity to infection

**DOI:** 10.1098/rsob.120075

**Published:** 2012-05

**Authors:** Ilias Kounatidis, Petros Ligoxygakis

**Affiliations:** Laboratory of Genes and Development, Department of Biochemistry, University of Oxford, South Parks Road, Oxford OX1 3QU, UK

**Keywords:** innate immunity, *Drosophila*, host defence

## Abstract

Innate immunity relies entirely upon germ-line encoded receptors, signalling components and effector molecules for the recognition and elimination of invading pathogens. The fruit fly *Drosophila melanogaster* with its powerful collection of genetic and genomic tools has been the model of choice to develop ideas about innate immunity and host–pathogen interactions. Here, we review current research in the field, encompassing all layers of defence from the role of the microbiota to systemic immune activation, and attempt to speculate on future directions and open questions.

## 2. Introduction

The study of *Drosophila* immunity was initiated at Umeå University, Sweden in the laboratory of microbiologist Hans Boman. In their seminal study, Boman *et al.* [1] clarified very early the humoral nature of the response, its inducibility and lack of specificity. It soon became apparent, however, that before the genetic backdrop of the response could be explored, it would be necessary to purify the factors responsible for this immune response. Because of its size *Drosophila* was not a good model in which to do this, so for the next 15 years Boman, co-workers and alumni of his research team started to investigate the giant silk moth *Hyalophora cecropia* opening the molecular era for the field of insect immunity (see [2,3] as examples of their work). Some of the tenants of this inducible immune reaction were found to be secreted antimicrobial peptides (AMPs), several classes of which were subsequently cloned and studied in several other species of Lepidoptera and Diptera (see [4] for review). It was still *Drosophila*, however, that gave the impetus to study in-depth defence reactions in insects and relate them to mammals. AMP gene promoters contained NF-*κ*B binding sites, crucial for their induction [5,6] and *Drosophila* Toll controlled AMP gene expression through NF-*κ*B [7]. Following this finding, the hypothetical receptors that Charles Janeway postulated being mediators of innate immunity were found to be homologues of Toll [8,9] a finding that not only re-defined the field of innate immunity as a whole, but also placed its evolution under a new perspective. Below, we attempt a current synthesis of *Drosophila* immunity highlighting its enormous progress as well as pinpoint some of the challenges that remain ahead.

## 3. Where does infection come from?

Like all organisms, insects live in a world containing an almost unquantifiable amount of micro-organisms. Some insects, however, are exposed considerably more than the average organism as they feed, lay their eggs and develop on decomposing media. These insects include *Drosophila* where part of its microbial load is introduced in the gut through the digestive process. Subsequently, a part of the digested microbes reach and may colonize the gastrointestinal epithelial wall. These micro-organisms may then become part of the commensal flora or induce pathogenicity and systemic immunity. In addition, systemic activation may occur through septic injury by nematodes or by wasps depositing their eggs on fruit fly larvae.

## 4. Epithelial responses and gut flora

Anatomically, the *Drosophila* gut can be divided into foregut, midgut and hindgut. The upper digestive system is used for food uptake and storage while processing and absorption takes place in the mid and posterior regions of the midgut. In this continuous system typical of higher Diptera, some of the meal is completely processed and defecated before some has even entered the digestive section of the midgut. The availability of gut-specific GAL4 lines combined with the advent of genome-wide RNAi libraries initiated the functional cell biology of the midgut (see below). It soon became apparent that the presence of intestinal stem cells (ISCs) ensures gut homeostasis with the supply of differentiated enterocytes (ECs). A characteristic of ECs is their rapid turnover where apoptotic cells are replaced by the compensatory proliferation of ISCs. ISCs were first described by the Spradling and Perrimon laboratories [10,11]. Similarly to mammals, the Notch, Wingless, platelet-derived growth factor (PDGF), epidermal growth factor (EGF), and insulin receptor pathways have been implicated in the maintenance, proliferation and/or differentiation of ISCs (see [12] for a review). In addition, Hippo signalling is used to restrict stem-cell proliferation in the gut of both *Drosophila* and mammals [13]. Recently, a controversy in the field was settled by recording the absence of active stem cells but presence of Wingless-expressing cells within the anterior pylorus, the proliferation of which provides homeostasis following serious damage [14].

In parallel to studies of gut physiology, intense investigation has been directed towards the elucidation of the *Drosophila* microbiota in both laboratory and field populations [15–20]. It was found that *Drosophila* is harbouring a community of gut bacteria that is much simpler compared with vertebrates and it is now possible to extract and cultivate these bacteria, use them in re-colonization experiments and produce mutants to interrogate host–pathogen interactions (see table 1). Combining functional cell biology and the knowledge of microbiota, several digestive infection models have been developed; these will be summarized below.

**Table 1.** Bacterial species associated with life stages of *D. melanogaster* from laboratory populations and collected from the wild.

bacterial genera	[15]	[17]	[18]	[20]	[19]	[16]
*Acetobacter*	+w, l	+l	+w	+l	+w, l	+l
*Acidovorax*			+w			
*Acinetobacter*			+w			
*Agrobacterium*			+w			
*Alcaligenes*			+w			
*Arcobacter*			+w			
*Azospirillum*						
*Bacillus*	+?					
*Bordetella*			+w			
*Bradyrhizobium*	+?					
*Chitinophaga*	+?					
*Citrobacter*	+?					
*Cladosporium*		+l				
*Commensalibacter*					+w, l	
*Corynebacterium*	+w					
*Dysgonomonas*					+w, l	
*Enterobacter*	+?		+w		+w, l	
*Enterococcus*	+w, l		+w		+w	
*Erwinia*	+?				+w	
*Frateuria*			+w			
*Gluconacetobacter*			+w	+l		
*Gluconobacter*	+?		+w			
*Klebsiella*	+?					
*Lactobacillus*	+?	+l	+w	+l	+w, l	+l
*Leuconostoc*	+?		+w			
*Morganella*	+?					
*Pantoea*	+?				+ w	
*Providencia*			+w		+w, l	
*Pseudomonas*	+?		+w			
*Serratia*	+?				+w, l	
*Shigella*					+w, l	
*Spiroplasma*					+w	
*Staphylococcus*	+?	+l			+w	+l
*Stenotrophomonas*	+?		+w			
*Vagococcus*						
*Weissella*	+?					
*Wolbachia*	+w		+w	+l	+w	

+, present in; l, laboratory strain; w, caught in the wild; ?, not specified; refs. [20,21] gut only, all other whole flies.

### 4.1. Commensal bacteria

The first observation of the possible role of flora to the development of *Drosophila* occurred more than 40 years ago. Bakula observed that axenic cultures of *Drosophila* larvae showed elongated developmental times [21]. Many years later, Brummel *et al*. [22] showed that the lifespan of adult flies under axenic conditions was reduced and that reintroducing bacteria during the first week of adult life could restore wild-type longevity. Bacterial flora seems to be necessary for optimal larval development upon nutrient scarcity. *Lactobacillus plantarum* is sufficient on its own to recapitulate the natural microbiota growth-promoting effect. *Lactobacillus plantarum* exerts its benefit by acting genetically upstream of the target of rapamycin (TOR)-dependent host nutrient sensing system controlling hormonal growth signalling [23].

Recently, Shin *et al.* [24] attempted to identify the molecular aspect of the above relationship between the development of the host and the flora. They showed the role of pyrroloquinoline quinone-dependent alcohol dehydrogenase (PQQ-ADH) of the commensal bacterium *Acetobacter pomorum* interacts with insulin/insulin-like growth factor signalling (IIS) in *Drosophila* to maintain the gut–microbe mutualism. The modulation of host IIS by the PQQ-ADH defines developmental factors like body size, energy metabolism and ISC activity of the host. Germ-free animals infected by PQQ-ADH-deficient bacteria showed deregulation of developmental and metabolic homeostasis. Both enhancement of the host IIS or enrichment of the diet with acetic acid (the metabolic product of PQQ-ADH) proved capable of reversing the above defects.

Studies above that determined the microbiota also showed the ability of commensal bacteria (like *L. plantarum*, *Lactobacillus brevis*, *A. pomorum*, *Enteroccocus faecalis*, Gluconobacter sp. and a bacterium in the family Acetobacteraceae, strain A911 of *Commensalibacter intestini*) to colonize germ-free adults [15–19,24]. In contrast, non-commensal bacteria like *Erwinia carotovora carotovora* and *Escherichia coli* did not exhibit the same capacity. Interestingly, the NF-*κ*B homologue Relish (see immune deficiency pathway below) was detected in the nucleus of intestinal cells in the presence of the microbiota [20]. The question was, therefore, how the host manages to maintain low levels of AMP and preserve the structure of its flora. Ryu *et al.* [20] showed that the intestinally expressed homeobox gene Caudal represses the NF-*κ*B-dependent AMP genes, in this way regulating commensal-gut homeostasis.

### 4.2. Non-commensal (pathogenic and non-pathogenic) bacteria

In 2000, the first natural bacterial infection of *Drosophila* larvae revealed the activation of host immune responses by different bacteria of the genus *Erwinia* [25]. It was the first time that systemic AMP production was recorded using an ingestion model. Importantly, the non-pathogenic strain *E. carotovora carotovora-15* (*Ecc-15*) has proved to be a valuable tool in exploring gut homeostasis. Tzou *et al.* [26], using the strain *Ecc-15*, showed that AMP production was following a tissue-specific pattern. For example, diptericin expression in larvae upon infection was observed in the proventriculus and part of the midgut, while no AMP expression was observed in this tissue. Foley & O'Farrell [27] showed the important signalling role of nitric oxide (NO) to innate immunity by using *Ecc-15* and *E. coli* in their feeding experiments. Nitric oxide synthase (NOS) was upregulated upon infection while its inactivation compromised host survival.

In their quest for a bacterium that can naturally infect and kill *Drosophila*, Bruno Lemaitre's laboratory isolated a previously uncharacterized bacterial species, *Pseudomonas entomophila* (*Pe*) that can orally infect and kill *Drosophila* larvae and adults [28]. The same group sequenced and assembled its genome [29] and interrogated *Pe* mutants for virulence factors [28,30,31]. From the side of the host, Vodovar *et al.* [28] showed the importance of an Immune deficiency (Imd)-dependent (see later for Imd signalling) local response against *Pe* as opposed to systemic immunity underlying the importance of local AMP expression against food-borne pathogens.

Using *Serratia marcescens* as a pathogenic bacterium Nehme *et al.* [32] confirmed the induction of both local and systemic immune responses and the importance of the consequent Imd-dependent local AMPs production to fight off infection. The availability of RNAi strains for more than 90 per cent of the *Drosophila* genome directed Cronin *et al.* [33] to follow a genome-wide *in vivo* RNAi screen revealing host genes involved in susceptibility or resistance to intestinal infection with *S. marcescens*. Applying whole-organism and tissue-specific knock down these authors uncovered that the JAK-STAT signalling pathway participated in intestinal defence by regulating stem cell proliferation. Participation of the JAK-STAT pathway along with Imd in gut immunity was also confirmed by conducting oral infections with *Ecc-15* [31]. This study showed that gut homeostasis includes inflection of the stress response, increased ISC proliferation and epithelia renewal in response to bacterial infection. Using *Pe*, Jiang *et al.* [34] showed that activation of JAK-STAT in ISCs was due to the production of cytokines (Upd, Upd2 and Upd3) by ECs in the midgut.

In addition, oral challenge by pathogenic bacteria revealed new information about the effects of the physical barrier of the peritopic matrix (PM), which lines the intestinal lumen. PM forms a layer of chitins and glycoproteins protecting the epithelium from rough food particles and microbes. Infection by *Ecc-15* showed that a gene for a putative eye-lens protein called drosocrystallin (Dcy) was strongly up-regulated upon infection but its expression was not controlled by the Imd pathway. The role of Dcy in adult PM formation was recently elucidated. Dcy-deficient flies showed an increased susceptibility to oral infections with the entomopathogenic bacteria *P. entomophila* and *S. marcescens* [35]**.**

Experiments in parallel with the above established ingestion models led to the identification of the important role of reactive oxygen species (ROS) in the gut immune response of *Drosophila*. Oral ingestion of bacteria induces the rapid synthesis of ROS in the gut by an NADPH oxidase called duox oxidase (DUOX). In cases of suppressed DUOX expression, an increased mortality rate upon minor infection in adults is recorded [36,37]. A signalling network that controls both positively and negatively the expression and activity of DUOX, important for the host response to commensal and pathogenic bacteria, was thus identified [38].

### 4.3. Fungi

Ingestion of *Cryptococcus neoformans* caused the death of the fly in contrast to the injection of *Saccharomyces cerevisae* or the nonpathogenic *Cryptococcus kuetzingii* or *Cryptococcus laurentii*. The Toll pathway did not show any role in *Drosophila* adult defense upon ingestion of *C. neoformans* [39]. However, Toll showed important roles to both clearance of *C. neoformans* cells and survival of adults after systemic infection by the yeast [39]. Recently, our laboratory developed a *Drosophila* model to study *Candida albicans* gastrointestinal (GI) infection [40]. *Candida albicans* GI infection caused extensive JNK-mediated death of gut cells and induced systemic activation of AMP activity in the larval fat body. Both phenomena were partially mediated through fungal proteases. From the side of the host, NO and blood cells influenced systemic AMP responses. The system is now ready for isolating both pathogen and host factors that influence gut pathogenesis and activation of systemic immunity.

The above, as well as parallel studies, have emphasized the integration of gut responses, blood cells and AMP systemic immunity in host defence through both paracrine and autocrine signals recently involving TGF-β signalling and tissue-specific regulation of AMPs by FOXO and Drifter/ventral veinless [41–43].

## 5. Layers of host defence in systemic immunity

### 5.1. Haemocytes

*Drosophila* counters systemic infection through the wide-ranging action of haemocytes, considered as the insect equivalent to vertebrate blood cells. Recent studies along with a classic paper by Hartenstein and colleagues [44] have delineated the ontogeny of these cells from embryonic development (plasmatocytes and crystal cells) to larval stages, where they persist and form circulating and sessile subpopulations, and then through metamorphosis to adults (for review, see [45]). Following the first phase of haematopoiesis in embryos, there is a second phase in larvae directed by a specialized compartmentalized organ situated in the dorsal aorta, namely the lymph gland (for review, see [46]). This organ contains progenitors (pro-haemocytes) for three types of functional haemocytes including the plasmatocytes, which are monocyte-like cells involved in phagocytosis of apoptotic bodies and pathogens, and crystal cells, which are required for melanization (see below). These two haemocyte types are released in the haemolymph upon dispersal of the lymph gland at the onset of the larva to pupa transition. The haematopoietic organ also gives rise to a third type of haemocyte, the lamellocyte, devoted to encapsulation of foreign bodies that are too large to be phagocytosed. Lamellocytes do not differentiate in normal developmental conditions but only in response to specific immune challenges such as wasp parasitism or stress conditions mediated by an increase of ROS. Mutant backgrounds with increased haemocyte proliferation lead to formation of ‘melanotic tumours’ that result from encapsulation of larval tissue by lamellocytes. In this context, large-scale screens to identify melanotic-tumour-suppressor genes have been published uncovering new genes and gene networks controlling haemocyte homeostasis [47–49].

One question that has long remained unanswered in the field was the possible interconnectedness of haemocyte responses to fat-body-directed AMP gene regulation. An early study proposed there was no such connection [50]. These results were based on the use of the *domino* (*dom*) mutant, which lacked more than 90 per cent of circulating haemocytes and a similar proportion of the sessile subpopulation [50]. Dom is a member of the SWI2/SNF2 family of DNA-dependent ATP-ases functioning as a global transcriptional regulator of proliferative tissues [51]. Larvae, carrying strong *dom* mutant alleles died in late larval/early pupal stages in the absence of infection and earlier when infected [51]. However, the experimental set-up precluded use of those early larvae including only those that survived immune challenge for measuring AMP gene expression, which was found to be comparable to wild-type larvae [50]. One additional caveat of the analysis was the general effect the mutation had on cell proliferation in many tissues other than haemocytes. Nevertheless, *dom* mutants failed to induce *diptericin* during Gram-negative GI infection [25], suggesting that blood cells could relay a signal emanating from the gut to activate the Imd pathway that controlled *diptericin* expression in the fat body. This signal may be NO as both bacterial and fungal GI infection need haemocytes to relay the NOS-generated signal to the fat body and induce systemic activation of AMP gene expression [27].

Additional evidence for the contribution of blood cells towards fat body antimicrobial responses came with the description of *psidin* by Brennan *et al*. [52]. Identified in a genetic screen for mutants with a reduced AMP response, *psidin* encodes a lysosomal protein required in haemocytes for degradation of engulfed bacteria as well as expression of the AMP gene *defencin* in the fat body, establishing thus a connection between pathogen detection by phagocytes and fat body AMP gene induction. This led to the proposition that haemocytes were internalizing and subsequently presenting non-self antigens to fat body cells [52], shifting the debate from whether there was a connection between haemocytes and fat body to whether the connection was antigen presentation or secreted signal(s). A problem with the Brennan paper, however, was that the rescue of the mutant with a wild-type copy of *psidin* was performed using *peroxidasin-GAL4*, which is also expressed in the fat body [53]. Therefore, it may be that *psidin* is needed in both tissues although the authors detected only expression of *psidin* in haemocytes [52].

Three studies published in 2009 redressed the debate by following a different approach. This was to genetically eliminate plasmatocytes by targeting their apoptosis through forced expression of pro-apoptotic genes [53–55]. It was found that haemocytes were indispensable for embryonic development [54] but surprisingly, their absence did not influence post-embryonic development [53–55]. This was interesting given the belief that haemocytes participated in extensive tissue remodelling during pupariation and reinforced the argument that larval lethality seen in *dom* and *psidin* mutants was not linked to blood cells but to other tissues. Haemocyte-ablated larvae were unable to mount a full systemic response following GI infection [53], while larval responses to systemic challenge were also dependent on the presence of haemocytes [53]. Silencing the Toll ligand *spz* in haemocytes produced the same result, namely, the significant reduction of Toll-dependent AMP responses [53]. Spz expressed by haemocytes could have both a paracrine as well as an autocrine function in AMP induction and is the first signal identified in the crosstalk between haemocytes and fat body in larvae. Evidence from a parallel study gave impetus to the idea of Spz as a pro-inflammatory cytokine in a feedback between haemocytes and fat body [56].

In contrast to larvae, absence of blood cells did not influence AMP gene induction in adults [54,55]. Haemocyte-deficient flies were significantly more susceptible to infection owing to the absence of phagocytosis, confirming early experiments which used latex beads to saturate the phagocytic machinery [54,55]. The fact that recent studies have shown that phagocytosis and AMP induction (through the Toll pathway, for example) had additive effects [57] but did not influence each other, indicated that, in adults, these are two independent systems which nevertheless act together to fight off infection. The idea, however, of the ‘internal milieu’ [58] and how immune homeostasis is indeed a result of metabolism interacting with other processes through secreted signals, has been explored in significant work implicating the effect of insulin signalling in *Mycobacterium marinum* infection [59]. In addition, recent work has shown that TGF-β signals emanating from specific subsets of adult haemocytes modulate infection-induced melanization and AMP gene expression in time [41]. The relation, therefore, between haemocytes and fat body in both larvae and adults remains an evolving picture.

### 5.2. Phagocytosis

**Figure 1.**
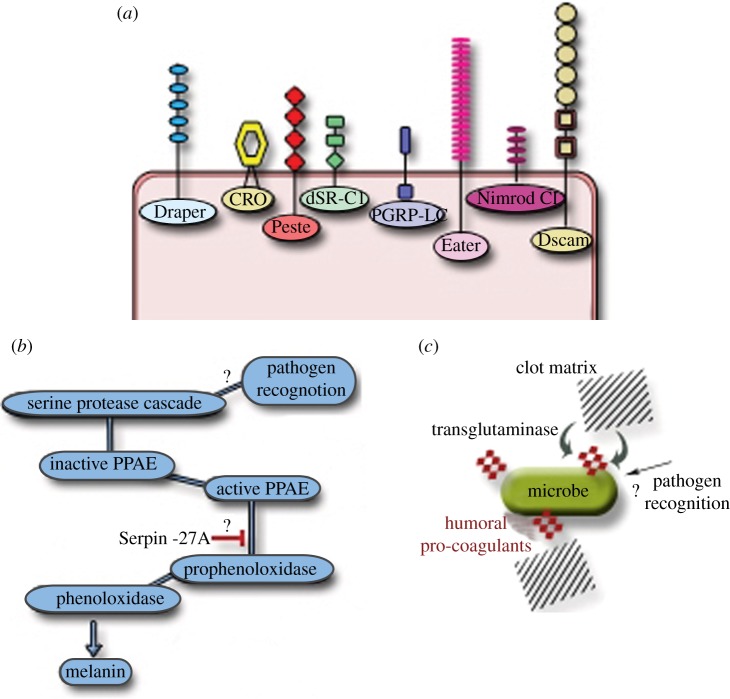
Layers of *Drosophila* immunity: (*a*) receptors found on the surface of *Drosophila* macrophages, (*b*) schematic of the melanization reaction and (*c*) coagulation. The link to pathogen recognition in both (*b*) and (*c*) still remains elusive. PPAE, pro-phenoloxidase activating enzyme.

One of the most powerful and immediate ways for fruit flies to eliminate apoptotic bodies, bacterial infection or fungal spores in the haemolymph is by their removal through receptor-mediated recognition and phagocytosis. *Drosophila* phagocytes have been used as a model for ‘professional’ mammalian phagocytosis (for review see [60]). This is because, during development, dead cells are recognized by evolutionary-conserved receptors such as Croquemort (CRO, the CD36 paralogue) [61] and Draper (the LPS recognition protein (RP) paralogue) [62], although the latter also recognizes lipoteichoic acid from *Staphylococcus aureus* and mediates uptake of this bacterium [63]. Studies of *Drosophila* S2 cells, which share many features with mammalian macrophages and are amenable to RNAi, identified phagocytic receptors relevant to host immunity, such as members of the scavenger receptor family Peste and dSR-C1 [64,65], peptidoglycan PGRP-LC [66], members of the Nimrod family of proteins Eater [67] and Nimrod C1 [68] and the IgSF-domain protein Dscam [69]. A summary of these receptors is schematically presented in figure 1*a*. However, the question of which components of the bacterial cell wall are recognized, and how, by these receptors is still open (for PGRP-LC see below). Nonetheless, significant advances have been made in the elucidation of intracellular signalling and actin regulation [70]. Measurements of time needed to eliminate pathogens by phagocytosis have resulted in describing an impressive capacity: systemically infected larvae with 3000 bacteria can eliminate almost 95 per cent of them in 30 min [53]. It is some hours later that AMP gene expression peaks and therefore a pertinent question was why larvae need AMPs at all. An interesting proposition came not from *Drosophila* but from *Tenebrio molitor* where the same time-course was observed in adults [71]. Rolf and co-workers proposed that the timing was crucial in order for AMPs to ‘meet’ a dramatically reduced number of bacteria and thus diminish the possibility for induction of resistance [71]. Moreover, their sustained expression and presence in the haemolymph long after the infection was cleared provided protective immunity.

### 5.3. Melanization

This is considered to be the earliest and most acute reaction of insects against pathogens breaching the cuticle and invading through septic injury. It is visible by the blackening of the wound site and the surface of the pathogen and is used to encapsulate and sequester pathogens too large to be phagocytosed, as seen with mosquito responses against the malaria parasite [72]. In addition, the intermediates of the reaction are directly toxic to microbes (for review, see [73]). In *Drosophila*, however, there was literature disputing the importance of melanization in fighting off infection [74,75]. Yet, a significant paper [76] showed elegantly through infection with various Gram-positive and Gram-negative bacteria, which induce strong systemic melanization in fruit flies, that melanization has a considerable impact on host survival following immune challenge. Knock down (or knock out) of one player in the proteolytic cascade leading to melanization (MP2; see below) was sufficient to significantly modulate survival after infection by either increasing susceptibility or augmenting tolerance [76]. Interestingly, even in the cases where there was no change in host survival there was a significant increase in bacterial load suggesting a different balance between resistance and tolerance [76]. An alternative interpretation of course could be that MP2 has roles additional to melanization as has been previously suggested [75].

Mechanistically, melanin synthesis is the final product of this proteolytic cascade involving the sequential activity of serine proteases MP1 and MP2, leading to the cleavage of prophenoloxidase (proPO) to phenoloxidase ([75]; see also figure 1*b*). The *Drosophila* genome encodes three proPOs, two expressed in crystal cells (*DoxA1* and *CG8193*) and one in lamelocytes (*DoxA3)* [77]. Activation of melanization is inhibited by Serpin-27A [78,79]. Although the target of Serpin-27A is thought to be prophenoloxidase activating enzyme as it inhibits the relevant beetle enzyme *in vitro* [79] the endogenous target of Serpin-27A is not known. An additional open question is the link between pathogen recognition and activation of the cascade. There is very detailed biochemical work in other insects (see [80,81]) but in *Drosophila*, where *in vivo* work is possible, these links have not been established.

### 5.4. Coagulation

An additional layer of innate responses to restrict pathogen dissemination from a wound is the process of haemolymph clotting. In the clot, various proteins form characteristic filaments which cross-link the bacteria and prevent their spread. Experiments following this early reaction *in vitro* indicated that initial clot formation was independent of melanization since it happened in proPO mutants [82]. *In vivo*, however, larvae lacking crystal cells had a reduced ability for clot formation and decreased capacity for wound healing [83]. These results showed that proPO may not be crucial for the formation of the clot *per se* but is important for the hardening of the larval coagulum as well as for healing a septic injury. Proteomic analysis has identified several proteins involved in clotting [84]. These proteins include Hemolectin, a large protein and a major component of the clot, produced by plasmatocytes [85]; the humoral pro-coagulants lipophorin, hexamerin and its receptor (also called fat body protein 1) [84]; Fondue, a haemolymph protein with its production regulated by Toll, which is not involved in initial clot formation but in cross-linking of clot fibers [86]; and Transglutaminase (TG), providing the connection between bacterial surfaces and the clot matrix [87]. TG binding was observed in a variety of bacterial surfaces although TG RNAi affected host survival in a limited number of infections [87]. The presence and role of TG, however, is widely conserved and has been shown to contribute to clot formation in almost every species where clotting has been studied in any detail (see [88] for review), suggesting that there might be qualitative differences in the binding of TG to different bacterial surfaces that ultimately produce differences in host survival. Whether the process of TG binding to microbial surfaces, which in turn aids clot matrix and pro-coagulant assembly to entrap pathogens, is connected to pathogen recognition is not yet clear. Conceptually, both microbial surface components and host–pathogen recognition receptors could serve as substrates for TG (summarized in figure 1*c*).

### 5.5. Fat-body-dependent antimicrobial peptide gene induction

Fat-body-dependent AMP gene induction, the hallmark of the systemic response, is the synthesis and secretion in the haemolymph of powerful effector molecules collectively known as AMPs. These are mostly small cationic peptides that directly attack the cell wall of microbes [4]. The cloning and characterization of their promoters paved the way to a series of now classic papers (see below) revealing the signalling pathways that controlled AMP gene expression, starting with the discovery that AMP gene expression was regulated by NF-*κ*B promoter elements (see [5,6] as examples of this work).

## 6. Signalling in systemic immunity

### 6.1. The Toll pathway

In contrast to its mammalian counterparts, *Drosophila* Toll is not activated by direct interaction with microbial molecules but through an endogenous ligand, namely the Nerve Growth Factor-related cytokine Spätzle (Spz) [89]. Binding is achieved by two Spz dimers, each interacting with the N terminus of one Toll molecule. This triggers a conformational change in what is now a dimeric Toll receptor, to activate downstream signalling [90]. Spz is in turn activated to bind to Toll via proteolytic cascades, which culminate in processing of its N-terminal pro-domain by the Spz-activating enzyme (SPE) [91]. It is still an open question whether the Spz pro-domain is separated from the hydrophobic C-106 domain when cleaved, as has been suggested *in vivo* [92], or remains attached through disulphide bonds, as seen in biochemical experiments, to be finally displaced when bound to Toll [93]. SPE is the point where pathogen recognition information is integrated through the activation of three recognition pathways: one triggered by fungal or bacterial proteases that directly activate the host serine protease Persephone [94,95], which in this context acts as a sensor of virulence [95,96]; one induced by recognition of fungal cell wall [96]; and one activated by Lysine (Lys)-type bacterial PG (see below). Both these last two recognition pathways converge to the modular serine protease (ModSP) [97], which in turn activates—not directly—the serine protease Grass [97,98]. Proteases Spirit, Spheroide and Sphinx1/2 were also identified as necessary for a host responding to both fungi and Gram-positive bacteria [98].

The recognition events that initiate the ModSP-Grass-SPE axis are mediated by two PGRPs, namely PGRP-SA and PGRP-SD and the glucan-binding protein GNBP1 [100,101]. These three molecules recognize Lys-type PG, a major component of Gram-positive bacteria [101]. Upon recognition, PGRP-SA and GNBP1 physically interact, forming a complex [101]. We have found that depending on the extent of PG cross-linking GNBP1 acts as an endomuramidase hydrolysing Lys-type PG with low cross-linking thus producing new glycan reducing ends, which are presented to PGRP-SA [102,103]. In contrast, Buchon *et al.* [97] suggested that full-length GNBP1 had no enzymatic activity. Crucially, however, these authors did not test the functionality of their recombinant GNBP1 in rescuing the relevant mutant, an important element when relating biochemical data to an *in vivo* hypothesis. Nevertheless, they suggested a (not mutually exclusive) role for GNBP1 as a linker between PGRP-SA and ModSP [97]. PGRP-SD functions as a receptor for Gram-positive bacteria with partial redundancy to the PGRP-SA–GNBP1 complex [98]. A pertinent question nevertheless is how a relatively small number of proteins recognize the vast variability in the cell wall of Gram-positive bacteria and how PG is even accessible as it is ‘buried’ under various cell-wall glycopolymers and bulky modifications. A strategy could be the use of more than one PGRP and/or various layers of different responses (see above) all linked to pathogen recognition.

Our results indicate that when accessibility to PG in the bacterial cell wall is not blocked by glycopolymers such as teichoic acids, then PGRP-SD becomes redundant [104]. It is interesting to note that when teichoic acids are not present in the bacterial cell wall the Toll pathway (but not PGRP-SA itself) becomes redundant as well, indicating that PGRP-SA has Toll-independent functions [104]. The glucan-binding GNBP3 is responsible for yeast recognition [96] and its N-terminal domain has been the only GNBP family of proteins with a crystal structure [105], revealing an immunoglobulin-like fold in which the glucan-binding site is masked by a loop. This loop is displaced during binding representing a novel mechanism for beta-glucan recognition [105].

**Figure 2.**
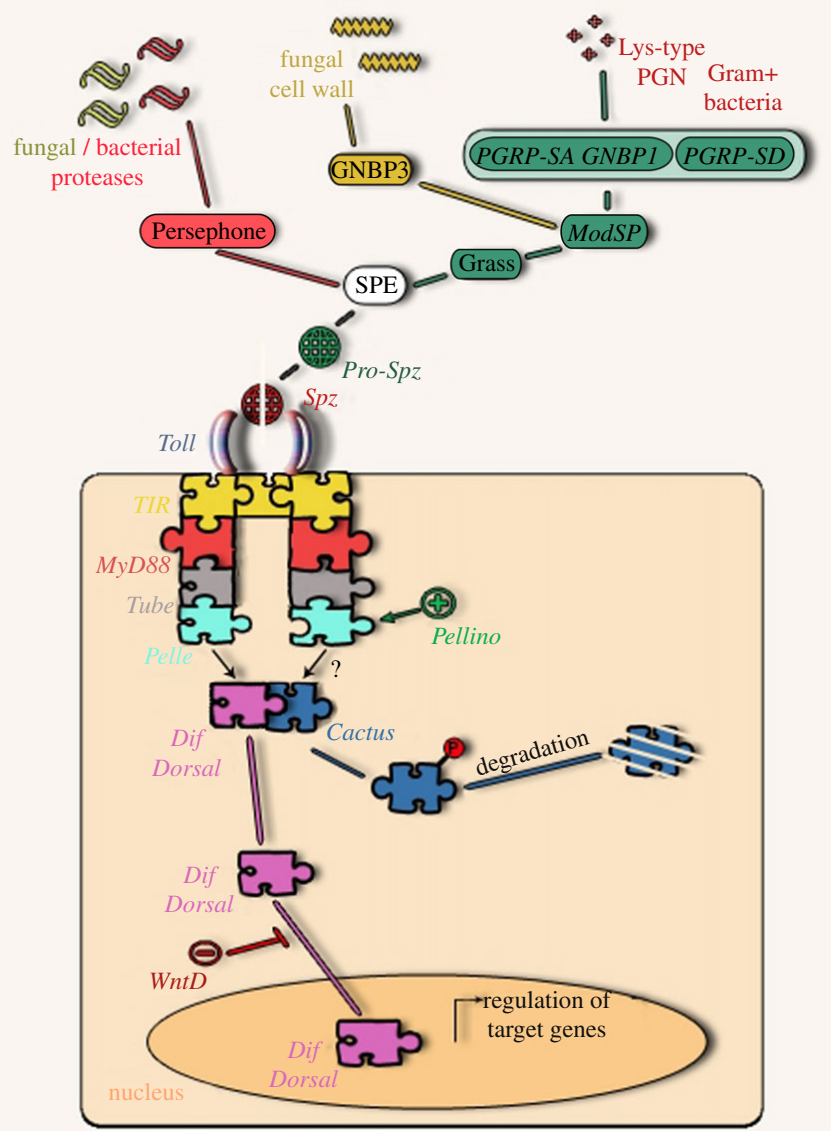
Summary of Toll signalling; see text for details.

Following Spz–Toll interaction a receptor–adaptor complex that will transmit the signal from the cell surface to the nucleus is formed. This complex comprises the MyD88 protein, which interacts with Toll through their respective Toll/Interleukin-1 receptor domains [106] and connects with Tube via death domain contacts that will in turn recruit the *Drosophila* IRAK homologue, the kinase Pelle [107]. The latter will directly or indirectly phosphorylate the IκB homologue Cactus, which is thus targeted for degradation. Upon Cactus degradation, the NF-*κ*B homologues Dorsal or Dif are free to move to the nucleus and regulate hundreds of target genes [108,109]. A positive regulator of the pathway is the RING-domain containing Pellino, acting presumably at the level of Pelle in parallel to mammalian Pellinos that modulate IRAK action [110]. In contrast, a negative regulator is WntD, which reduces Toll activity by preventing translocation of Dorsal to the nucleus [111]. In addition, it has recently been shown that endocytosis is paramount for efficient Toll signalling [112]. A schematic summary of Toll pathway signalling is presented in figure 2.

### 6.2. The immune deficiency pathway

**Figure 3.**
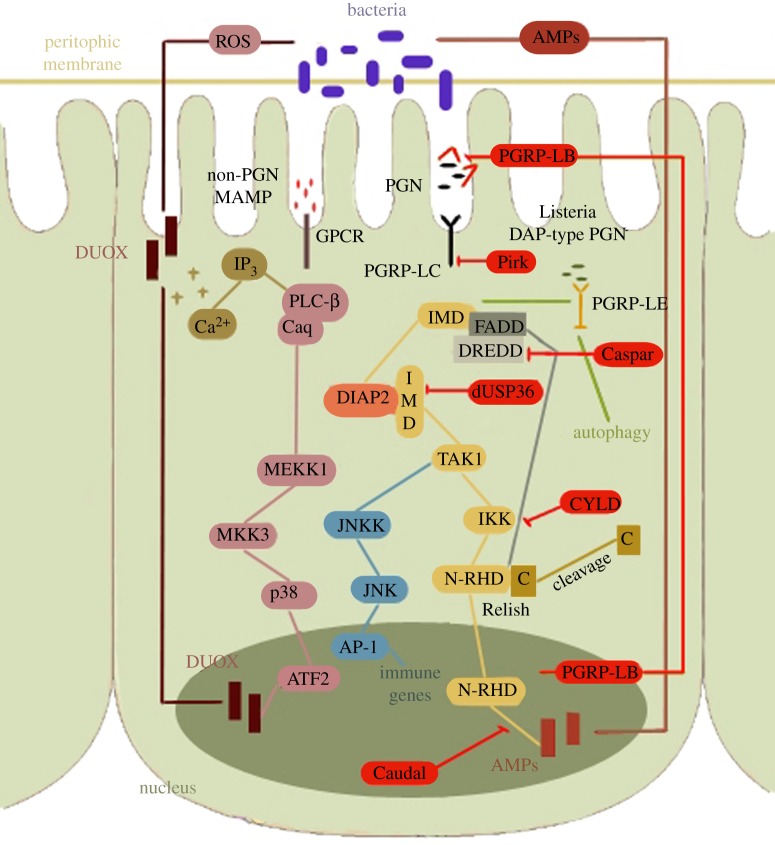
Summary of gut defences and Imd signalling; see text for details.

In addition to Toll there is another pathway, which is primarily activated by DAP-type bacterial PG, namely the immune deficiency (IMD) pathway (see figure 3 for summary of both systemic signalling and network of gut defences). DAP-type PG forms the cell wall of Gram-negative bacteria as well as some Gram-positive *Bacilli* [113]. Pathogen recognition in IMD occurs through the transmembrane PGRP-LC and the intracellular PGRP-LE [114,115]. PGRP-LC is a type-2 transmembrane receptor, with an extracellular PGRP domain that is critical for recognizing extracellular bacteria, while PGRP-LE lacks a transmembrane domain and functions as an intracellular receptor, although an extracellular cleaved form of PGRP-LE made only of the PGRP domain has also been reported in cell culture [114].

Flies deficient in both PGRP-LC and -LE are unable to induce AMPs in response to Gram-negative bacteria, being highly susceptible to these infections [113,114]. PGRP-LC encodes three receptors via alternative splicing, namely PGRP-LCx, PGRP-LCy and PGRP-LCa [116]. All three proteins share the same intracellular signalling domain while the extracellular recognition part is unique for each receptor variant [116]. In contrast to PGRP-LCy, whose role remains unclear, it is well established that the other two PGRP-LC splice forms have important functions in activating IMD. On its own, PGRP-LCx is sufficient to respond to *E. coli* PG whereas both PGRP-LCx and PGRP-LCa form a heterodimer upon recognition of a monomeric disaccharide–tetrapeptide fragment of PG known as tracheal cytotoxin (TCT) [114]. With an as yet unknown mechanism, TCT is able to enter cells and is then sensed by PGRP-LE. This interaction induces the formation of head-to-tail homo-oligomers of PGRP-LE [117]. In addition, PGRP-LE acts as recognition receptor for intracellular bacteria such as *Listeria monocytogenes*. In this case, PGRP-LE induces autophagy through an IMD-independent pathway [118] in keeping with the ability of *Listeria* to trigger such responses in mammalian epithelial cells [119].

Subsequent intracellular signalling is transduced through the RHIM-like motif found in PGRP-LC and -LE [114,115]. However, the molecular mechanism by which the RHIM-like domains in PGRP-LC and -LE regulate signalling is unclear. A protein, which binds both LC and LE, is Imd itself, a death-domain-containing protein with homology to mammalian RIP1 (minus the kinase domain) [120]. In turn, Imd associates with the *Drosophila* FADD (FAS-associated death-domain protein) homologue via a homotypic death-domain interaction [121]. FADD then recruits and interacts with the homologue of mammalian caspase-8, apical caspase death-related Ced-3/Nedd2-like protein (DREDD) [122], via the death-effector domains found in these proteins [123,124]. It is not known whether recruitment of DREDD to the PGRP–IMD–FADD complex is sufficient for its activation.

DREDD cleaves Imd thus unmasking a domain of interaction of the latter with the *Drosophila* Inhibitor of apoptosis-2 (dIAP-2) [125]. In its turn, dIAP-2, through its RING domain, ubiquitinates and stabilizes Imd, which then acts as a scaffold for the recruitment of downstream components. It is conceivable that the ubiquitin-specific protease 36 (dUSP36) acts to suppress the pathway by reversing this ubiquitination [126]. Components downstream of Imd are TAK1 [127] and its adaptor TAB2 [128]. It is not yet shown whether TAK1 is recruited in an Imd complex but this seems to be the working hypothesis [125]. Once recruited, TAK1 would trigger activation of the IκB-Kinase (IKK) complex, which in turn phosphorylates the NF-*κ*B protein Relish [129]. Relish is a composite protein made of a C-terminal IκB domain and an N-terminal NF-*κ*B part [130]. DREDD is the most probable protein that mediates Relish cleavage resulting in the uncoupling of two Relish domains, thereby allowing the N-terminal to translocate into the nucleus [129,131]. Although Relish phosphorylation is dispensable for its cleavage, it appears to enhance the activity of Relish as a transcription factor in the nucleus [129]. Separately, TAK1 also activates the JNK kinase, which initiates the phosphorylation and nuclear translocation of the transcription factor AP-1 [132].

As mentioned earlier, the Imd pathway is also involved in gut infection. In this context, a number of negative regulators (both intra- as well as extracellular) have been identified. These include the secreted PGRP-LB [133], which has an amidase catalytic activity cleaving DAP-type PG, limiting availability of ligand for PGRP-LC and thus dampening the Imd signal. Inside cells, a protein interacting with PGRP-LC, namely Pirk, has been shown to negatively regulate the Imd pathway not only in the gut but also during systemic activation [134–136]. Flies lacking Pirk exhibited higher levels of AMPs although a resolution of the response was still observed. However, double *pirk;PGRP-LB* mutants resulted in a further increase showing the synergy of those two factors to control gut defenses [137]. Additionally, the three members of the PGRP-SC locus negatively regulate the pathway in systemic mode [137] and triple *pirk;PGRP-SC;PGRP-LB* mutants (where the whole PGRP-SC locus has been deleted) showed low viability and a level of AMPs that was 8 times higher at 24 h post infection compared with wild-type flies [137]. The triple mutant also had compromised life span even in unchallenged conditions suggesting that persistent activation of the pathway (presumably mediated by the gut flora or by ingested bacteria) was deleterious [137]. Another negative regulator of the pathway suggested to act at the level of DREDD is a homologue of the Fas-associated factor FAF-1 [138].

Caspar-deficient flies upregulate AMPs in the absence of immune challenge and are more resistant to bacterial infections [138]. An additional intracellular negative regulator of the Imd pathway is Cylindromatosis (CYLD), probably at the level of IKK [139]. It is intriguing that every step of the intracellular part of the pathway has its own negative regulator; until now only TAK1 has been devoid of such a partner, although POSH has been identified as a protein limiting the amount of activated TAK1 and thus restricting the timing of the JNK branch of the TAK1 signal [140].

## 7. Emerging complexities in *Drosophila* immunity

The Toll–Imd pathway dichotomy that, as a (very powerful) working hypothesis, has dominated the field for the best part of the 1990s and early 2000s has run its course. There is well-documented evidence of cross-reaction by using elicitors that were traditionally thought as triggers of only one pathway [141–143]. In addition, through genome-wide screening in S2 cells, an array of new genes that influence expression of Toll-dependent or Imd-dependent AMP gene expression have been identified, although their relationship to the core pathways remains to be explored [144–146].

It has also become increasingly obvious that different pathogens elicit different host response *strategies*, which although dependent on the two pathways and many defences described above, have a connection to physiology and behaviour. Insulin signalling, food uptake and circadian rhythms [59,147,148] have been found to have a significant effect on host survival in parallel to mammalian models. These results have certainly introduced a holistic view of host defence as part of the life history of the organism, while introducing (through the study of microbiota) an ecological perspective that was absent during the intense years of gene discovery. In addition, host responses to viral infection induce RNAi and involve JAK/STAT signalling [149–152]. However, the measure of involvement of the latter pathway has not been tested using all available mutants. Finally, both the Toll and Imd pathways have been implicated in antiviral responses [153,154].

## 8. Outlook

Far from being a ‘fill-in-the-blanks’ exercise after the positioning of the pathways and systems involved, *Drosophila* immunity has been used as a model for wide-ranging biology and continues to be so. The directions of study on the interaction of the microflora with the host are endless and tap on any number of physiological/developmental issues [23] and recently even mating [155]. Results are fascinating, especially in parallel to the human microbiome project as *Drosophila* can be a much simpler organism. At the same time, the host–pathogen interaction aspect at the molecular level is the one that has not been systematically explored. We know a lot about the host reaction but do not know enough about how this reaction is altered when the pathogen changes. So a systematic genome-wide exploration of pathogen mutants and their interaction with fruit fly immunity is important. An additional aspect that has not been explored sufficiently is interaction with *natural* parasites, despite some early efforts on the subject [156–159].

Finally, the elephant in the room: the hallmark of vertebrate responses is memory, which shapes the almost absolute specificity of the defence. Insects have many of the characteristics of vertebrate immunity (discrimination of self versus non-self, amplification and dissemination of defences throughout the body) but seem to lack the more sophisticated aspects of immunological memory. Or do they? There has been evidence of some form of memory in insects since the beginnings of the field in the classic work of Metalnikow [160]. One much more recent report in *Drosophila* studied memory following infection by *Streptococcus pneumoniae* [161] and found that fruit flies better survived lethal doses of the microbe when a previous challenge with the same pathogen ‘primed’ them. However, what it is specifically with *S. pneumoniae* that provokes a memory response (or whether this is a more general phenomenon) remains to be determined.

Future exploration of *Drosophila* immunity on the open questions above and beyond them will generate exciting biology revealing new aspects in the evolution and regulation of host defences.
